# Knowledge and Awareness of Breath-Holding Spells in Infants and Toddlers Among Parents in Saudi Arabia

**DOI:** 10.7759/cureus.77703

**Published:** 2025-01-20

**Authors:** Kholoud A Hothan, Reem S Al Judaibi, Mona S Alshabi, Raghad H Alkhalifah, Nouf A Alturki, Shahad Shawish, Rawan M Alsofyani, Tahani F Alanazi, Ahmed K Bamaga, Mohamed A Ali, Nadia M Fida

**Affiliations:** 1 Pediatrics, Faculty of Medicine, King Abdulaziz University, Jeddah, SAU; 2 Pediatrics, College of Medicine and Surgery, Batterjee Medical College, Jeddah, SAU; 3 Pediatrics, College of Medicine and Surgery, Taibah university, Medina, SAU; 4 Pediatrics, College of Medicine, Qassim University, Qassim, SAU; 5 Pediatric Residency, Ministry of National Guard Health Affairs, Jeddah, SAU; 6 Pediatric Residency, Alhada Armed Force Hospital, Taif, SAU; 7 Medicine, Faculty of Medicine, University of Tabuk, Tabuk, SAU; 8 Pediatric Neurology, Faculty of Medicine, King Abdulaziz University, Jeddah, SAU; 9 Pediatrics, Faculty of Medicine, King Abdulaziz University, Cairo, EGY

**Keywords:** breath-holding spells, child health, healthcare education, parental knowledge, public health

## Abstract

Background and objective

Breath-holding spells (BHS) are common in infants and young children, often triggered by emotional factors like anger or frustration. While their exact cause remains unknown, potential causative factors include iron deficiency and delayed brain development. Despite their frequency, many parents lack proper awareness and understanding of BHS. This study assessed parental knowledge and awareness of BHS among Saudi Arabian parents of infants and toddlers.

Methods

A cross-sectional study was conducted using an online questionnaire from November 1, 2023, to April 30, 2024. Participants were Saudi Arabian adults with at least one child. The questionnaire was validated for reliability and validity. Data were collected on sociodemographic characteristics, parental knowledge of BHS, and experiences with the condition. Data were analyzed using SPSS Statistics 24.0 (IBM Corp., Armonk, NY), with Chi-square tests employed to assess relationships between variables. Ethical approval was obtained, and informed consent was acquired from all participants.

Results

A total of 251 participants were included; while most parents were female and well-educated, many lacked awareness of BHS. Emotional triggers were common causes, and inappropriate responses, such as shaking, occurred frequently. Around a quarter of parents reported experiencing BHS with their children. Knowledge regarding BHS was significantly higher among those parents who had firsthand experience with BHS or those with older children.

Conclusions

Parents in our sampled cohort had limited knowledge and awareness of BHS. Many parents lack a proper understanding of appropriate management strategies and often resort to harmful practices. These findings emphasize the need for targeted educational interventions to improve parental knowledge and promote safe and effective management of BHS.

## Introduction

Breath-holding spells (BHS) are known as paroxysmal non-epileptic episodes, which are usually provoked by a triggering event such as anger or frustration [1]. There are two primary types of BHS: cyanotic and pallid. In cyanotic BHS, a child will usually cry intensely for a short period and then abruptly cease crying and hold their breath until they become cyanotic and lose consciousness. They may also become sweaty, experience body jerks, or lose bladder control. Fortunately, these episodes are short-lived, and the child usually regains consciousness without any intervention; however, the child may seem sleepy for a while [2]. Cyanotic BHS is the most common type of BHS. On the other hand, pallid BHS occurs in response to painful or frightening experiences. During the episode, the child’s heart rate slows down and the child stops breathing, loses consciousness, and turns pale [3]. There is also a mixed type that exhibits features of both cyanotic and pallid BHS [4]. BHS affects 5% of otherwise healthy infants and young children aged six months to six years, and most cases of BHS occur before the child reaches 18 months of age [5]. Both males and females are equally affected, with a slight male predominance [6,7]. 

While the precise etiology of BHS is not fully understood, several potential factors have been identified, Iron deficiency anemia or iron deficiency alone has been linked to these episodes, and correcting iron deficiency has shown promise in reducing their frequency. Also, an imbalance between antioxidants and oxidants and deficiencies of trace elements such as zinc, iron, and selenium may contribute to autonomic dysfunction leading to BHS. According to a case-control study, a delay in the maturation of brainstem myelination could play a role in the etiology of BHS in children [8]. There is likely right-to-left shunting of blood across the foramen ovale during a Valsalva maneuver, which may occur during a breath-holding episode. Intrapulmonary shunting caused by a mismatch in ventilation and perfusion or flow through bronchopulmonary or other arteriovenous anastomoses may also be a contributing factor. Furthermore, it can be triggered by emotional factors such as fear and anger; some children may use this behavior as a means of seeking attention, and they return to their normal state after getting what they want [9]. 

Previous research on BHS has primarily explored the potential causes and management of these conditions; however, there is a lack of studies on parental awareness of the condition. Therefore, we aim in this study to assess parental knowledge and awareness of BHS. We hypothesize that many parents have a lack of information or are mostly misinformed about BHS and, consequently, are not aware of the appropriate actions to be taken during these events. Knowledge and awareness of BHS among parents are key as these would play a huge role in reassuring parents. This cross-sectional study aims to determine the level of awareness and knowledge of BHS in infants and toddlers among parents in Saudi Arabia.

## Materials and methods

Study design

A cross-sectional study was conducted in Saudi Arabia to assess parents' knowledge and awareness of BHS in infants and toddlers. The study lasted six months, from November 1, 2023, to April 30, 2024.

Participants

The inclusion criteria were as follows: adults of both genders aged 18 years or older, living in Saudi Arabia, with at least one child, and who completed the survey. Individuals younger than 18 years, those who had not experienced parenthood, and those who did not complete the survey were excluded from the study. Out of 308 participants, 251 fully completed the questionnaire.

Questionnaire

The questionnaire, hosted on a secure Google Forms platform, consisted of three parts - part A: sociodemographic characteristics of the participants; part B: assessment of parents' knowledge and awareness of BHS; and part C: experience with BHS, including how parents managed them. Before its distribution, the questionnaire was pre-tested on 20 parents to assess validity and reliability. The Breath-Holding Spells Questionnaire showed excellent internal consistency, with a Cronbach’s alpha of 0.786. Construct validity was supported by a strong positive correlation with parents' self-reported knowledge (r=0.63), and criterion validity was demonstrated by a significant relationship with children's medical history of BHS (r=0.54).

Data collection

Data were collected using an online questionnaire distributed through social media platforms (X, Telegram, WhatsApp) across different regions of Saudi Arabia.

Ethical considerations

Ethical aspects were comprehensively addressed during the study. Approval was secured from the Institutional Review Board (IRB) at the Ministry of Higher Education, King Abdulaziz University Faculty of Medicine Research Ethics Committee, Jeddah, Saudi Arabia (reference no. 638-23).

Statistical analysis

Statistical analysis was performed using SPSS Statistics 24.0 (IBM Corp., Armonk, NY). Results were presented as mean and standard deviation (SD) for quantitative data, frequencies, and percent for qualitative data. The knowledge score was calculated, then the knowledge percentage was calculated by dividing the obtained score by the maximum score, and then the product by 100. The mean score was then compared according to different factors by using the student t-test when comparing between two groups and ANOVA when comparing between more than two groups. A p-value of less than or equal to 0.05 was considered statistically significant.

## Results

A total of 251 parents were included in this study; 83 (33.1%) belonged to the age group of 31-40 years. The majority were females, (213, 84.9%), and most participants had a bachelor's degree (182, 72.5%). A large majority were married (288, 90.8%) and the children were predominantly male (137, 54.6%). More than half of the children were aged three years (140, 55.8%). More than half of the participants (152, 60.6%) were from the Western region. The demographics of the participants are presented in Table 1.

**Table 1:** Sociodemographic characteristics

Characteristics		Frequency (n)	Percentage (%)
Age of the parent, years	18–20	12	4.8%
	21–30	73	29.1%
	31–40	83	33.1%
	41–50	66	26.3%
	51–60	12	4.8%
	61 and above	5	2.0%
Gender of the parent	Female	213	84.9%
	Male	38	15.1%
Level of education	High school	34	13.5%
	Middle school	14	5.6%
	Master’s degree	16	6.4%
	Bachelor’s degree	182	72.5%
	Doctorate	5	2.0%
Marital status	Divorced	8	3.2%
	Married	228	90.8%
	Single	10	4.0%
	Widowed	5	2.0%
Gender of your child	Female	114	45.4%
	Male	137	54.6%
Age of your child, years	1	60	23.9%
	2	51	20.3%
	3	140	55.8%
Where is your residence in Saudi Arabia?	Central region	28	11.2%
	Eastern region	41	16.3%
	Northern region	22	8.8%
	Southern region	8	3.2%
	Western region	152	60.6%

The knowledge of the parents was assessed through seven questions (Table 2). Less than half of the participants (120, 47.8%), had heard about BHS, and the largest proportion of those, 60 (23.9%) reported having a basic understanding of the same. Emotional status, such as frustration, was the major reported cause of BHS by parents (143, 57%). Shaking the child (79, 31.5%) was the most commonly reported action when witnessing a BHS by patients, followed by starting CPR (64, 25.5%) and splashing water on the face of the child (62, 24.7%). Only 34 (13.5%) believed that this condition is benign, whereas 196 (78.1%) thought that it is a curable condition. The largest proportion (93, 37.1%) of the parents were not sure that this condition has long-term sequelae in the child. More than one answer was allowed for some questions.

**Table 2:** Knowledge of the parents regarding BHS BHS: breath-holding spell; CPR: cardiopulmonary resuscitation

Question		Frequency (n)	Percentage (%)
Have you ever heard about breath-holding spells?	No	131	52.2%
	Yes	120	47.8%
If yes, how would you describe your understanding of breath-holding spells?	Advanced	8	3.2%
	Basic	60	23.9%
	Moderate	52	20.7%
What are the possible causes of breath-holding spells?	Abnormal electrical activity of the brain	27	10.7%
	Emotional states such as frustration	143	57.0%
	Genetic causes	20	7.9%
	Incomplete development of the brain	46	18.3%
	Iron deficiency	54	21.5%
	Spiritual causes such as Envy, Evil Eye, and Magic	15	6.0%%
	others	12	4.8%
What do you think you should do when witnessing a breath-holding spell?	Shake the child	79	31.5
	Splash water on the child’s face	62	24.7
	Start CPR	64	25.5
	Stay calm and do nothing	56	22.3
	Transfer the child immediately to the hospital	85	33.9
Do you believe a breath-holding spell is a benign condition?	No	122	48.6%
	Not sure	95	37.8%
	Yes	34	13.5%
Do you think this condition is curable?	No	8	3.2%
	Not sure	47	18.7%
	Yes	196	78.1%
Do you think this condition has long-term sequelae on the child?	No	68	27.1%
	Not sure	93	37.1%
	Yes	90	35.9%

The practices of parents who had witnessed the condition in their children (n=70) were investigated via nine questions (Table 3). The largest proportion of parents reported that their children were in the age group of one to two years when experiencing a brief period of stopping to breathe (36, 51.4%). Most parents (51, 72.9%) reported less than one minute as the duration of these episodes, and the major symptoms experienced by the child were blushing and discoloration of lips and extremities (54, 77.1%). Most parents reported they had witnessed BHS only once (27, 38.6%), followed by less than five times (26, 37.1%). The main trigger of the condition reported by the parents was emotional (51, 92.9%); however, only 34 (48.6%) consulted a pediatrician or healthcare professional for such episodes. More than one-half (39, 55.7%) reported that they would trust the doctor’s opinion if the doctor explained that the condition was normal and there was no need for any investigation or treatment. Many parents (69, 98.6%) stated that their children did not develop seizures or were not diagnosed with epilepsy, and they do not have a positive family history of epilepsy (64, 91.4%). More than one answer was allowed for some questions.

**Table 3:** Practices of those who had witnessed BHS in children (n=70) BHS: breath-holding spell

Questions		Frequency (n)	Percentage (%)
How old was your child when experiencing a brief period of stop breathing?	I don't remember	6	8.6%
	Less than one year	21	30.0%
	1-2 years	36	51.4%
	3-4 years	3	4.3%
	5-6 years	3	4.3%
	More than 6 years	1	1.4%
What was the duration of the episode?	Less than one minute	51	72.9%
	More than one min	11	15.7%
	Don’t remember	8	11.4%
Which of these symptoms did your child experience during the episode?	Bluish discoloration of lips and extremities	54	77.1%
	Abnormal movement of the extremities	13	18.6%
	Unconsciousness	13	18.6%
	Jaw locking	7	10%
	Tongue biting	1	1.4%
	Nausea	1	1.4%
	Vomiting	8	11.4%
How many times has your child experienced breath-holding spells?	One	27	38.6%
	Less than five	26	37.1%
	More than five	8	11.4%
	I don't remember	9	12.9%
What was the trigger of these episodes?	Emotional triggers (fear, anger, sadness, etc.)	51	92.9%
	Others (please specify)	6	7.1%
	Pain at any site of the body	9	12.9%
	Head trauma	12	17.1%
Have you consulted a pediatrician/healthcare professional for these episodes?	No	36	51.4%
	Yes	34	48.6%
If the doctor explained to you that your child is normal and does not need any investigation/treatment/medications, would you ask for another opinion?	Not sure	5	7.1%
	No, I'll seek another opinion by consulting another doctor	25	35.7%
	No, I'll seek another opinion by asking family and friends	1	1.4%
	Yes, I'll trust in the doctor's opinion	39	55.7%
Has your child ever developed seizures or been diagnosed with epilepsy?	No	69	98.6%
	Yes	1	1.4%
Do you have a positive family history of epilepsy?	No	64	91.4%
	Yes	6	8.6%

The correlations between the mean score related to knowledge and the demographics of the participants are shown in Table 6. Two significant correlations were found: between the mean score related to knowledge and experiencing a BHS in children (p=0.02); and between the mean score related to knowledge and the age of children (p=0.018).

**Table 4:** Correlation between knowledge score and demographic characteristics *Statistically significant SD: standard deviation

Characteristics		Mean	SD	P-value
Age of the parents, years	18–20	36.6667	18.74874	0.274
	21–30	31.5068	20.79471	
	31–40	31.0843	22.36134	
	41–50	25.1515	15.81065	
	51-60	26.6667	15.56998	
	61 and above	44.0000	35.77709	
Gender of the parent	Female	31.0526	21.15445	0.730
	Male	25.8824	20.61445	
Education	High and middle school	25.8824	20.61445	0.254
	Bachelor’s degree	31.0989	20.21708	
	Master's and doctorate	28.0000	20.69385	
Marital status	Single	30.0000	14.14214	0.9
	Married	30.0877	20.08772	
	Widowed/divorced	27.6923	28.91189	
Gender of the child	Female	31.2281	21.12501	0.440
	Male	28.9051	19.69170	
Experience a breath-holding spell	Yes	35.1429	21.38284	0.020*
	No	27.9558	19.62759	
Age of the child, years	1	31.6667	19.92925	0.018*
	2	36.0784	20.40377	
	3	27.0000	20.06106	

## Discussion

The purpose of this cross-sectional study is to assess the knowledge and awareness of BHS among parents of infants and toddlers in Saudi Arabia by using a validated, online-based questionnaire. Parental awareness of BHS is a topic that has not received enough attention in previous research; prior studies on the condition have mostly focused on possible causes and therapeutic strategies. In our study, we included 251 parents, and only 27.9% of the parents reported having witnessed their children experience BHS. In a previous study conducted in Turkey involving 933 children, 3.4% were found to have BHS [10]. Another study conducted in Saudi Arabia with 602 parents revealed that 22.9% had previously witnessed at least one episode of BHS. Compared to the study conducted in the Makkah region of Saudi Arabia, our research showed a higher percentage of BHS; this difference may be due to the smaller number of participants in our study relative to the other study [11].

Of note, 47.8% of our participants had heard about BHS, 23.9% had a basic understanding, and the majority believed that BHS primarily occurs due to emotional factors like frustration (57%), while 21.5% thought that it was caused by iron deficiency anemia. Leung et al. found that several factors can lead to BHS, including iron deficiency anemia, cardiac inhibition due to parasympathetic hyperactivity, autonomic nervous system dysfunction, and brainstem myelination delay [12]. In the study by Abuaish et al., most of the participants (407 parents) believed that iron deficiency anemia could cause the spells, while 116 parents thought that frustration, anger, and pain could contribute to BHS events [11]. In our study, the second possible cause, after emotional factors, was iron deficiency anemia. Arslan et al.'s study to assess the effect of iron therapy in 100 children with BHS, regardless of hemoglobin levels, concluded that iron therapy reduces the frequency of spells, regardless of anemia, in all children with BHS. They recommended that all children with spells receive three months of empirical iron therapy [13]. The same recommendation was made by Hancı et al., who included 136 children with BHS, aged 1-48 months, in their study to assess the response to iron therapy. They found that 52 (39%) responded completely with complete remission [14].

On the other hand, 33.9% of the parents believe the best action to take when one of their children experiences BHS is to bring them to the hospital immediately, followed by shaking the child (31.5%), starting CPR (25.5%), staying calm and doing nothing (25.3%), and splashing water on the child's face (24.7%). According to the Children's Hospital in Philadelphia, the best action to take when a child experiences a BHS episode is to lay the child flat on the floor, avoid shaking or slapping, ensure the airway is clear, blow on the child's face, apply a cold, damp cloth to their face, and not start CPR if the child is not conscious. Furthermore, they should call for help if the child does not respond after two minutes, as it may not be BHS [15]. To the best of our knowledge, no research has been conducted to assess parental actions regarding BHS, which limits our ability to draw correlations from other studies. Further research is needed to determine the level of parental awareness of the management of BHS.

A high number of our participants had good knowledge about BHS and how it could occur, as well as when they should consult healthcare professionals. However, the majority had not heard about it before; hence, we need to raise more awareness about what BHS is, how it can happen, and what should be done when it occurs. One of the powerful ways to discuss topics nowadays is through social media, especially YouTube. Demirtas and Alici conducted the first study examining videos about BHS on YouTube. They evaluated 55 videos, and their study concluded that the majority contained useful information of sufficient quality. They highlighted the importance of collaboration between universities and academic institutes to produce better-quality videos [16]. We highly recommend the production of BHS videos in Arabic, as the majority of videos currently available on YouTube are in English.

Although 122 of the parents thought that BHS was not a benign condition, and 95 of the parents were unsure if it was benign or not, 196 parents believed it was a curable condition. The study by Abuaish et al. showed similar findings to our study, as most parents (90%) believed that BHS is a dangerous condition [11]. This indicates that most parents remain anxious about BHS, although the majority consider it a treatable condition. Of note, 51.4% of parents did not consult a pediatrician or healthcare worker when their children experienced their first episode of BHS, while 48.6% sought medical advice. This split may suggest varying levels of concern or awareness among parents about BHS. However, 55.7% of our participants indicated that they trusted the doctor’s opinion if he explained to them that there was no need for medical intervention and that it was normal. Hence, we need to raise more awareness about how benign the condition is, as the overall knowledge and attitudes regarding the protection of children and adolescents in Saudi Arabia have significantly increased as a result of the educational campaign for caregivers [17].

In our study, 27.9% of the parents responded affirmatively when asked if their children had ever experienced a BHS. In comparison to the study conducted in the Makkah region, which reported that 22.9% of parents witnessed their children experience BHS, our study revealed a higher percentage [11]. The difference could be attributed to our study being conducted in various cities in Saudi Arabia, while the other study was confined to the Makkah region, as well as the possibility of a higher frequency in regions other than Makkah.

In our study, among 70 parents whose children developed BHS, 51.4% witnessed incidents occurring between the ages of one and two years, and 72.9% lasted for less than a minute. The highest proportion of children (38.6%) developed BHS only once or fewer than five times (11.49%). In comparison to the study from the Makkah region, which included 602 participants who reported the first episode of BHS at the age of 15 months [11], 77.1% of children experienced BHS in the form of bluish discoloration of the lips and extremities. To the best of our knowledge, no research has been done to assess parents’ actions toward BHS, and thus we cannot establish correlations with other studies. On the other hand, 92.9% reported that the episodes were triggered by emotional factors such as fear, anger, and sadness. Additionally, about 12.9% linked the episodes to pain at any site in the body, and 17.1% associated them with head trauma. This highlights that while emotional factors are predominant, physical factors can also play a significant role. As for the study conducted in the Makkah region, the emotional factor triggers represent 67.6% [11].

Our research offers valuable insights into BHS awareness among Saudi Arabian citizens; however, the study has a few limitations. Our study's online platform and convenient sampling technique may restrict its generalizability. The focus on self-reported information may have led to reporting bias, which could be influenced by participants' attitudes, understanding of the items, or tendency to express their experiences in specific ways. Finally, our cross-sectional study sample consisted of only 251 participants, which may affect the generalizability and statistical power of the research.

## Conclusions

This study assessed Saudi Arabian parents' knowledge and awareness of BHS in their children. The results revealed that less than half of the parents were aware of BHS, and many lacked a comprehensive understanding of how to manage these episodes. Frequently reported inappropriate responses included shaking the child and initiating CPR. Even though most parents were aware of the possible risks associated with BHS, there was significant uncertainty regarding its long-term effects and management. These findings highlight the need for focused educational initiatives to improve parents' knowledge of and responses to BHS and ensure that these incidents are managed more safely and efficiently.

## Figures and Tables

**Figure 1:**
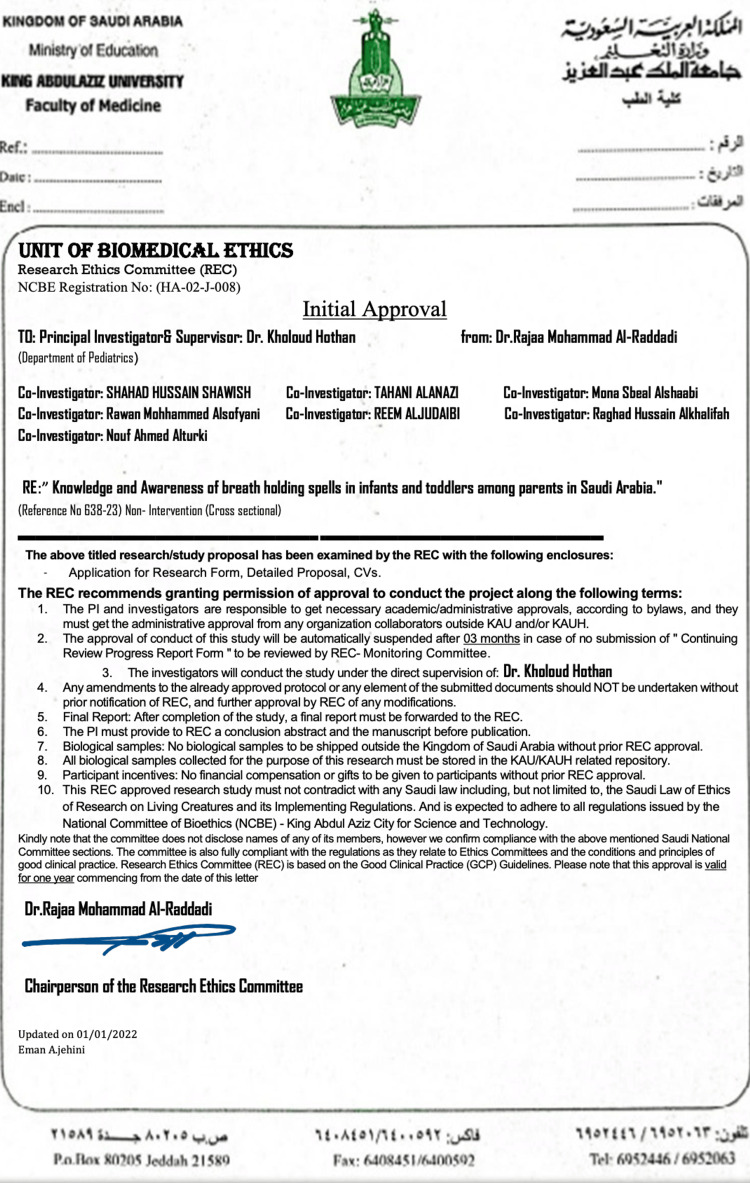
Institutional Review Board approval letter

## Appendices

**Table 5:** Questionnaire

General questions	
Question	Options
Do you have children?	• Yes • No
If yes, how many children do you have?	• (Open-ended)
Age of the parents:	• 18-20 • 21-30 • 31-40 • 41-50 • 51-60 • 61 and above
Gender of the parent:	• Male • Female
Level of education:	• Middle school • High school • Bachelor’s degree • Master’s degree • Doctorate
Marital status:	• Single • Married • Divorced • Widowed
Gender of your child:	• Male • Female
Age of your child (in years):	• 1 year • 2 years • 3 years
Where is your residence in Saudi Arabia?	• Eastern Region • Western Region • Northern Region • Central Region • Southern Region

Breath-holding spells - awareness	
Question	Options
Have you ever heard about breath-holding spells?	• Yes • No
If yes, how would you describe your understanding of breath-holding spells?	• Basic • Moderate • Advanced
What do you believe are the possible causes of breath-holding spells?	• Iron deficiency • Emotional states (frustration, anger, fear, pain) • Incomplete brain development • Abnormal electrical brain activity • Genetic causes • Spiritual causes (envy, evil eye, magic) • Others
If 'Others,' please specify:	• (Open-ended)
What do you think you should do when witnessing a breath-holding spell?	• Start CPR • Shake the child • Splash water on the child’s face • Transfer the child immediately to the hospital • Stay calm and do nothing
Do you believe breath-holding spells are a benign condition?	• Yes • No • Not sure
Do you think this condition is curable?	• Yes • No • Not sure
Do you think this condition has long-term sequelae on the child?	• Yes • No • Not sure
Has your child ever experienced a breath-holding spell (brief period of not breathing)?	• Yes • No
If yes, how old was your child during the episode?	• Less than one year • 1-2 years • 3-4 years • 5-6 years • More than 6 years • I don’t remember
What was the duration of the episode?	• Less than one minute • More than one minute • I don’t remember
Which symptoms did your child experience during the episode?	• Bluish discoloration of lips and extremities • Tongue biting • Jaw locking • Loss of bladder control • Abnormal movements of the extremities • Unconsciousness • Nausea • Vomiting
How many times has your child experienced breath-holding spells?	• One time • Less than five times • More than five times • I don’t remember
What was the trigger for these episodes?	• Emotional triggers (fear, anger, sadness, etc.) • Head trauma • Pain at any site • Others
If 'Others,' please specify:	• (Open-ended)
Have you consulted a pediatrician/healthcare professional for these episodes?	• Yes • No
If the doctor said no investigation/treatment is needed, would you ask for another opinion?	• Yes, I’ll trust the doctor’s opinion • No, I’ll consult another doctor • No, I’ll ask family and friends • Not sure
Has your child ever developed seizures or been diagnosed with epilepsy?	• Yes • No
Do you have a positive family history of epilepsy?	• Yes • No
